# Experiences of care partners and residents with the Long-Term Care Palliative Toolkit during the COVID-19 pandemic: A multiple methods study

**DOI:** 10.1177/26323524251393344

**Published:** 2025-11-12

**Authors:** Marie-Lee Yous, Rose McCloskey, Abigail Wickson-Griffiths, Donny Li, Vanessa Maradiaga Rivas, Amit Arya, Sheila A. Boamah, Maureen Dobbins, Pamela Durepos, Paulette V. Hunter, Sarah Neil-Sztramko, Henry Siu, Tamara Sussman, Genevieve Thompson, Sharon Kaasalainen

**Affiliations:** 1 School of Nursing, McMaster University Faculty of Health Sciences, Hamilton, ON, Canada; 2 Department of Nursing and Health Sciences, University of New Brunswick Saint John, NB, Canada; 3 Faculty of Nursing, University of Regina, SK, Canada; 4 Michael G. DeGroote School of Medicine, McMaster University Faculty of Health Sciences, Hamilton, ON, Canada; 5 McMaster University, Hamilton, ON, Canada; 6 Faculty of Nursing, University of New Brunswick, Hanwell, NB, Canada; 7 Department of Psychology, St. Thomas More College, Saskatoon, SK, Canada; 8 Department of Health Research Methods Evidence and Impact, McMaster University, Hamilton, ON, Canada; 9 Department of Medicine, McMaster University, Hamilton, ON, Canada; 10 School of Social Work, McGill University, Montreal, QC, Canada; 11 University of Manitoba, Winnipeg, MB, Canada

**Keywords:** conferences, education, end-of-life, care partners, healthcare providers, long-term care, older adults, palliative care, virtual care, palliative approach

## Abstract

**Background:**

With a large burden of suffering and death in 2020 due to COVID-19 in long-term care (LTC) homes resulting in restrictions of visitations, there is a need for a formal virtual intervention to support families/friends (i.e., care partners) and residents around palliative care, including planning for end-of-life when outbreaks like these occur. The LTC Palliative Toolkit includes informational resources for care partners, residents, and healthcare providers about the trajectory of life-limiting chronic illnesses (i.e., frailty, dementia, heart failure, kidney disease, lung disease) and Palliative Care Conferences (PCCs).

**Objective:**

To evaluate the impact of the LTC Palliative Toolkit on preparedness for end-of-life and satisfaction with information and to explore the experiences of care partners and residents with the virtual delivery of the components of the LTC Palliative Toolkit (i.e., informational pamphlets and PCCs).

**Methods:**

A multiple methods design was employed. Three LTC homes, one from each province (Ontario, New Brunswick, and Saskatchewan, Canada), were selected to reflect diverse contexts (e.g., ownership, staff turnover, facility size, and location). Caring Ahead surveys focusing on actions, dementia knowledge, communication, and emotions and support needs were conducted with care partners before and after PCCs to evaluate how prepared they felt about their relative or friend’s end-of-life and their satisfaction. Some care partners and residents completed telephone semi-structured interviews to explore their experiences with care received.

**Results:**

Survey findings revealed only one statistically significant improvement after the intervention period, an improvement in the emotion and support needs domain following PCCs, with baseline and follow-up mean scores of 6.08 (SD = 1.06) and 6.35 (SD = 1.16), respectively (*p* = 0.016). Qualitative interviews identified that the LTC Palliative Toolkit was a valuable intervention for both care partners and residents.

**Conclusion:**

The LTC Palliative Toolkit is suitable for use in any context and demonstrated high acceptability during the COVID-19 pandemic.

## Introduction

As of July 26, 2025, over seven million deaths worldwide were attributed to the COVID-19 pandemic, including over 55,000 deaths in Canada.^1^ In the first wave of the pandemic in Canada, which took place from March to August 2020, residents living in long-term care (LTC) homes accounted for approximately 81% of COVID-19-related deaths compared to an average of 38% in other countries.^2,3^ The high death rate in Canada during the first wave was attributed to pre-existing issues in its LTC homes such as chronic understaffing, lack of staff education, and inadequate funding.^3^ These factors were compounded by the vulnerability of the LTC population to the COVID-19 virus due to shared space, advanced age, a weak immune system, and the likelihood of having multiple comorbidities.^4^ With more deaths in LTC, there was a greater demand for increased awareness and integration of a palliative approach to care.^5^ More than ever before, in 2020, advanced care planning and goals of care discussions involving interdisciplinary team members, families/friends (i.e., care partners), and residents became an important priority in Canadian LTC homes.^5,6^

A palliative approach to care supports a seamless transition from moving into LTC through to the end-of-life. It begins with advance care planning to activate critical and early communication with residents and families so that they can be prepared for future decisions, including those regarding end-of-life care.^7^ A successful palliative approach to care informs and prepares both regulated and unregulated staff, and care partners and residents for end-of-life decision-making.^8,9^ We have demonstrated that interdisciplinary palliative champion teams improve team functioning and sustainability of palliative programs within LTC^10,11^ and Comfort Care Rounds offer staff dedicated time to debrief following a death.^12^

The COVID-19 pandemic exacerbated existing barriers to implementing a palliative approach to care for residents and care partners as they were more often faced with making critical and emotional end-of-life decisions without any preparatory discussions. In LTC, decision-making barriers include high rates of cognitive impairment among residents, prognostic uncertainty, and lack of continuity of care, limiting involvement in discussions with LTC staff and their care partners.^13,14^ Barriers to implementing a palliative approach to care include lack of training for staff, including unregulated staff such as personal support workers (PSWs), who provide the majority of direct resident care.^13,14^ During the first wave of the COVID-19 pandemic, limitations placed on resident activity and movement accelerated rates of frailty and cognitive decline, staff education pauses, staff shortages, and increase in acutely ill people, effectively increased barriers to a palliative approach.^15^

Although life expectancy for Canadian LTC residents is about 2 years,^16^ most LTC homes lack a formal palliative program.^17^ Hence, our research team developed the Strengthening a Palliative Approach in Long-Term Care (SPA-LTC) program, which has been informed by a scoping review of the literature^18^ and a stakeholder analysis.^19^ The program is currently being evaluated in 18 homes across three provinces (Ontario, Manitoba, and Saskatchewan). The program includes multiple evidence-informed components: (a) an interdisciplinary champion team (to provide leadership and support implementation); (b) Comfort Care Rounds with staff (for capacity building, reflection, and grief support); (c) informational pamphlets to help prepare residents and families about what to expect as their condition/disease progresses; (d) Palliative Care Conferences (PCCs) with care partners, interdisciplinary staff, and residents if able to attend to discuss changes in health and planning for end-of-life care; and (e) post bereavement follow-up for residents, care partners, and staff.

Pilot findings of the SPA-LTC program, prior to COVID-19 revealed promising results, including: (a) a 55% reduction in emergency room visits and 21% decrease in unnecessary emergency room visits at end-of-life^20^; (b) an increase in comfort with discussing advance care planning reported by 67% of residents and care partners^21^; and (c) an implementation rate of 82% of pre-death PCCs. Care partners also perceived that PCCs supported individualized, holistic care as well as ensured that there was a mutual agreement of care plans among care partners and staff.^20^ We decided to implement a virtual version of two key SPA-LTC components to support care partners with a palliative approach to care during the COVID-19 pandemic as face-to-face interventions cannot always be offered. The virtual version was called the LTC Palliative Toolkit.

### Virtual LTC Palliative Toolkit

The Virtual LTC Palliative Toolkit was based on the original SPA-LTC program and was implemented during the COVID-19 pandemic in LTC homes in three provinces (Ontario, Saskatchewan, and New Brunswick). The toolkit was evaluated by care partners and residents and included the following components: (a) *Virtual Informational Pamphlets* that focused on five life-limiting chronic illnesses, which are most prevalent in LTC (i.e., frailty, dementia, heart failure, kidney disease, and lung disease) as well as grief and bereavement. The pamphlets provided information about the expected illness trajectory and solicited questions from care partners and residents with links to videos and websites, and (b) *PCCs (in person and virtual)* that included discussions around changes in health and planning for end-of-life care with care partners, residents, and interdisciplinary staff such as nurses, PSWs/healthcare aides, physicians, physiotherapists, occupational therapists, recreational therapists, social workers, and dieticians. The PCC lasted for about an hour. Residents were invited to join as well if they were able to follow discussions. Care partners were provided with a virtual or hard copy of the Comfort Care at end-of-life booklet, an informational resource about a palliative approach for advancing dementia and other neurological diseases. This informational resource was intended to help care partners be more informed about End-of-Life (EOL) issues as well as think through questions for the conference. Although these components have been evaluated in previous studies, they have not yet been implemented as virtual interventions.^20,21^ The virtual PCCs can be a practical alternative during a pandemic through adherence to public health restrictions or requirements, which limits in-person visitation. The virtual pamphlets were readily available to care partners and residents through a website and specific links.

Considering the demonstrated benefits of a more formal palliative approach to care program in LTC and the challenges brought on by COVID-19, the purpose of this multiple methods study was to evaluate the impact of the LTC Palliative Toolkit on preparedness for end-of-life and satisfaction with information received. Unlike most advanced care planning interventions that focus on clinicians or residents directly, this study evaluated resources designed specifically for care partners of residents in LTC. A secondary objective was to explore the experiences of families and residents with specific elements of the LTC Palliative Toolkit (i.e., informational pamphlets and PCCs) offered in a virtual format. The research questions were: (a) What are the effects of the PCCs on care partners’ preparedness for end-of-life; (b) How satisfied are care partners with information received from the informational pamphlets; and (c) What are the experiences of residents and care partners with the LTC Palliative Toolkit delivered virtually during the COVID-19 pandemic (April 2021–January 2023) in LTC homes?

## Methods

### Study design

A multiple methods design was employed to address the research questions.^22,23^ The Mixed Methods Reporting in Rehabilitation and Health Sciences (MMR-RHS) checklist was used as a reporting guideline (see Supplemental Appendix).^24^ This checklist was considered the best fit to a multiple methods design.

### Setting, sample, and recruitment

One LTC home from each of the three Canadian provinces (Ontario (central Canada), New Brunswick (eastern Canada), and Saskatchewan (western Canada)) were selected as intervention sites to enhance the diversity of contexts found in LTC homes across Canada (e.g., ownership (private vs not-for-profit), staff turnover, facility size (i.e., 50–150 residents), location (i.e., rural vs urban)). The sample size for the number of participants completing surveys and interviews was determined based on our previous SPA-LTC work, the explorative nature of this study, and our access to these LTC homes.^20^ Specifically, we aimed to include a minimum of 20 care partners from each site for the surveys, and a minimum of 10 care partners and 10 residents from each site for the interviews. Care partners who completed the survey questionnaires could also complete the interview. Care partners and residents were recruited through posters located in the LTC homes, word of mouth, and by LTC staff who inquired about their interest in participating in the study if they met the study eligibility criteria. Convenience sampling was used to recruit participants. Participants were eligible to participate if they were a resident or a care partner at one of the three LTC sites and were able to speak, read, and write in English. Residents needed to be cognitively able to respond to questions and did not need to have a care partner participate in the study. Once care partners and residents agreed to participate in the study, a research assistant obtained consent and asked them to complete the Caring Ahead survey^25^ to measure family preparedness for end-of-life before and after completing the PCC.

### Data collection

Survey and interview data were collected from April 2021 to January 2023. Each LTC home collected data within their own timeframe and took part in the study for 6 months. Only one care partner per resident was recruited. Care partners completed the Caring Ahead survey^25^ at the beginning of the study prior to the PCC and at the completion of the 6-month intervention in Ontario and immediately following the PCC in Saskatchewan. Homes were asked to support research activities to the extent feasible in a pandemic context, which led to some homes not participating in specific survey collections and interviews due to resource constraints. For example, the home in New Brunswick did not complete PCCs and the Caring Ahead survey was therefore not administered to participants. The Caring Ahead survey is intended to measure and assess feelings of preparedness for end-of-life in care partners of persons with dementia and includes four domains (actions, dementia knowledge, communication, and emotions and support needs); internal consistencies of subscales were strong (Cronbach alphas ranged from 0.78 to 0.86).^25^ Items were scored on a Likert-type scale from 1 (strongly disagree) to 7 (strongly agree) with higher scores representing stronger agreement. The survey was validated and pilot tested among a sample of current care partners.

Care partners were also invited to complete a survey to evaluate bereavement pamphlets and other informational pamphlets that were sent by a link via email, immediately after reviewing these. These care partners did not necessarily need to have completed the PCC to review the pamphlets. Care partners were asked to complete a survey that explored the presentation, clarity, and usefulness of the pamphlets. These items were scored on a Likert-type scale from 1 (strongly disagree) to 5 (strongly agree). The survey used to evaluate the bereavement pamphlets was developed internally by the researchers and had not been validated or pilot tested due to time constraints. The questions used to evaluate the informational pamphlets were developed using the System Usability Scale, which has been extensively validated through decades of research.^26^ Surveys were completed electronically through a link sent by email connected to an online survey on the RedCap software or by telephone with the assistance of a research assistant. Demographic data collected for both care partners and residents included age and sex. None of the family members in Saskatchewan completed the survey assessing the pamphlets due to resource constraints. See Supplemental Files for surveys.

At the 6-month mark after the intervention was introduced, residents and care partners participated in telephone interviews ranging from 18 to 60 min to explore their experiences with care. All interviews were conducted by a research assistant who used an interview guide that was developed after a review of the literature for concepts such as a palliative approach, end-of-life care, COVID-19, and LTC. The semi-structured guide included questions about care partners’ perceptions of the care provided to residents since admission and, if applicable, during a COVID-19 outbreak in the home. Questions regarding the adequacy of information received, support in decision-making, and respect for one’s wishes were explored.

### Data analysis

Descriptive analyses of participants’ characteristics and survey results of pamphlets were expressed as a mean (standard deviation (SD)) for continuous variables and count for categorical variables. Pre-Post Palliative Care Conference-Caring Ahead survey data underwent a normalcy assessment using Shapiro–Wilk tests to determine whether the data were normally distributed. This assessment helped to inform the decision to use parametric or non-parametric tests to assess the statistical significance of the change in scores from baseline to 6 months in Ontario and immediately after the conferences in Saskatchewan for the measures of effectiveness. Based on the findings, Wilcoxon signed-rank tests were performed. Quantitative data analyses were conducted using SPSS version 29.

The qualitative software program NVivo^27^ was used to organize and analyze individual interview data. A thematic analysis of interviews was conducted.^28^ Key concepts were developed from the data using inductive coding and categorizing. Codes that were similar were grouped together under a theme. Two investigators (M.Y. and R.M.) independently analyzed and reviewed the data to enhance credibility and dependability. Data analysis was conducted in an iterative manner until consensus was reached among M.Y. and R.M. through discussion. These members have expertise in a palliative approach, LTC, and dementia. Several methods were used to improve the credibility of the findings (i.e., data triangulation of data sources and investigators, regular team meetings occurring every 3–4 months with the leads and co-investigators). Qualitative data were combined for all three sites.

### Ethical considerations

Ethics approval was granted from each provincial site, including the Hamilton Integrated Research Ethics Board (#12972) in Ontario, University of New Brunswick (#012-2021), and the University of Regina (#2021-031) in Saskatchewan. All participants received a written introduction to the study and an informed consent form written in lay language. Participants provided verbal or written consent. They provided verbal consent by meeting with a research assistant over the telephone or written consent in person at the LTC home.

## Results

### Quantitative analysis

#### Demographics

A total of 43 participants took part in the study with most being care partners (n = 41; 95%). Of these, 19 were from New Brunswick, 14 from Ontario, and 8 from Saskatchewan. The majority of care partners were female (n = 33) and between the ages of 51 and 70 (n = 27). With regards to resident participants, one resident was female, and the other was male. Both residents had been living in LTC for approximately 3 years. See Table 1 for participant demographics.

**Table 1.** Care partner and resident demographics.

Category	*n* (%)		
Care partner			
Site	ON (*n* = 14)	NB (*n* = 19)	SK (*n* = 8)
Sex			
Male	2 (14.3)	5 (26.3)	1 (12.5)
Female	12 (85.7)	14 (73.7)	7 (87.5)
Age in years (mean [SD])	64.4 [6.9]	58.5 [15.5]	66 [9.8]
31–40	0	4 (21.1)	0
41–50		1 (5.3)	0
51–60	6 (42.9)	5 (26.3)	2 (25)
61–70	6 (42.9)	3 (15.8)	3 (37.5)
71–80	2 (14.3)	5 (26.3)	3 (37.5)
81+	0	1 (5.3)	0
Relationship to resident			
Spouse	2 (14.3)	2 (10.5)	2 (25)
Child	11 (78.6)	11 (57.9)	5 (62.5)
Sibling	0	1 (5.3)	1 (12.5)
Other (e.g., grandchild, cousin, niece, nephew, friend, neighbor)	1 (7.1)	5 (26.3)	
Number of years resident living/lived in LTC (mean [SD]) — ON and SK only	2.8 [2.0]	N/A	4.1 [1.5]
Number of years resident living/lived in LTC home included in the study (mean [SD])	2.8 [2.0]	2.8 [2.9]	4.1 [1.5]
Resident (participants who took part in interviews; *n* = 2 — ON)			
Sex			
Male	1 (50)		
Female	1 (50)		
Age in years (mean [SD])	78.5 [12.0]		
70–80	1 (50)		
81–90	1 (50)		
Number of years living in LTC (mean [SD])	3.3 [0.4]		
Number of years living in LTC home included in the study (mean [SD])	3 [0]		

LTC: long-term care; ON: Ontario; NB: New Brunswick; SK: Saskatchewan; SD: standard deviation; N/A: not available.

The total counts do not add up to 100% due to rounding.

#### Family preparedness for end-of-life

At baseline, families in Ontario and Saskatchewan reported that they perceived that they were well prepared for end-of-life with regards to each of the domains on the Caring Ahead tool (i.e., dementia knowledge, communication, actions, emotion and support needs, and overall preparedness) with mean scores ranging from 5.9 (SD = 1.13) to 6.5 (SD = 0.55). The only statistically significant improvement found at the post-intervention period was in the emotion and support needs domain, with baseline and follow-up mean scores of 6.08 (SD = 1.06) and 6.35 (SD = 1.16), respectively (*p* = 0.016). See Table 2 for detailed results.

**Table 2.** Pre-Post Palliative Care Conference-Caring Ahead survey results.

Domain	Baseline mean (SD) – *n* = 18 (*n* = 11 — ON; *n* = 7 — SK)	Post-conference mean (SD) – *n* = 12 (*n* = 6 — ON; *n* = 6 — SK)	*Z*	*p* Value
Dementia knowledge (items 1–5)	6.50 (0.55)	6.68 (0.45)	0.857	0.391
Communication (items 6–9)	6.21 (0.97)	6.14 (1.32)	0.000	1.000
Actions (items 10–16)	5.93 (1.13)	6.16 (1.21)	0.848	0.396
Emotions and support needs (17–20)	6.08 (1.06)	6.35 (1.16)	2.410	0.016*
Overall preparedness (single item)	6.12 (0.99)	6.17 (0.94)	0.577	0.564

ON: Ontario; SK: Saskatchewan; SD: standard deviation.

Two-sided Wilcoxon signed-rank tests were used. Items are scored on a Likert-type scale from 1 (strongly disagree) to 7 (strongly agree).

* Denotes a *p* value<0.05.

#### Bereavement and informational pamphlets

Family members in Ontario and New Brunswick perceived that the bereavement pamphlets provided helpful information and were easy to understand. Informational pamphlets focused on chronic diseases were only reviewed in Ontario. These virtual pamphlets were found to be easy to access and easy to read. See Tables 3 and 4 for ratings.

**Table 3.** Bereavement pamphlets.

Pamphlet	Score (mean (SD))	
	ON (*n* = 4)	NB (*n* = 19)
Grief and loss		
The information in this pamphlet was presented clearly	4.5 (1)	4.5 (0.5)
The information in this pamphlet was easy to understand	4.5 (1)	4.6 (0.5)
The information in this pamphlet was upsetting or distressing	1.5 (0.6)	2.5 (0.9)
The information in this pamphlet was helpful	5 (0)	4.5 (0.7)
The information in this pamphlet was meaningful and relevant	4.8 (0.5)	4.6 (0.5)
The information in this pamphlet was reassuring	4.5 (0.6)	4.3 (0.7)
The pamphlet had sections that I felt were not important	1.5 (0.6)	2.3 (0.8)
The pamphlet provided very helpful resources	4.3 (1)	4.5 (0.6)
Some of the information in this pamphlet was new to me	2.8 (1.5)	3.4 (1.3)
Resources on grief, bereavement, and loss		
The information in this pamphlet was presented clearly	5 (0)	4.6 (0.5)
The information in this pamphlet was easy to understand	5 (0)	4.5 (0.5)
The information in this pamphlet was upsetting or distressing	1 (0)	2.4 (1.2)
The information in this pamphlet was helpful	5 (0)	4.4 (0.8)
The information in this pamphlet was meaningful and relevant	5 (0)	4.4 (0.6)
The information in this pamphlet was reassuring	5 (0)	4.4 (0.6)
The pamphlet had sections that I felt were not important	2 (0)	2.4 (1.1)
The pamphlet provided very helpful resources	3 (0)	4.5 (0.5)
Some of the information in this pamphlet was new to me	1 (0)	3.5 (1.1)
What to do after a death		
The information in this pamphlet was presented clearly	5 (0)	4.4 (1)
The information in this pamphlet was easy to understand	5 (0)	4.6 (0.5)
The information in this pamphlet was upsetting or distressing	1(0)	2.5 (1.3)
The information in this pamphlet was helpful	4 (0)	4.3 (0.7)
The information in this pamphlet was meaningful and relevant	5 (0)	4.6 (0.6)
The information in this pamphlet was reassuring	4 (0)	4.2 (0.7)
The pamphlet had sections that I felt were not important	1 (0)	2.4 (1.2)
The pamphlet provided very helpful resources	4 (0)	4.2 (1)
Some of the information in this pamphlet was new to me	3 (0)	3.4 (1.4)
All pamphlets		
Reading the pamphlets made me feel supported	4 (1.2)	4.4 (0.7)
Reading the pamphlets helped me to understand my feelings	4.3 (1)	4.3 (0.7)
Reading the pamphlets helped me to feel help was available if needed	4.3 (0.5)	4.6 (0.6)
Reading the pamphlets helped to clarify the actions I may need to take	4.3 (1)	4.5 (0.6)
Reading the pamphlets helped to clarify where I can go for additional support	4.3 (0.5)	4.7 (0.5)

ON: Ontario; NB: New Brunswick.

Items are scored on a Likert-type scale from 1 (strongly disagree) to 5 (strongly agree).

**Table 4.** Informational pamphlets (*N* = 4 — ON).

Question	Score (mean (SD))
I think that I would like to use the virtual pamphlet frequently	2.5 (0.6)
I found the virtual pamphlet unnecessarily complex	1.5 (0.6)
I thought the virtual pamphlet was easy to read	4.3 (1.0)
I think that I would need the support of a technical person to be able to access the virtual pamphlet	1.0 (0)
I would imagine that most people would learn to access the virtual pamphlet very quickly	2.0 (1.0)
I found the virtual pamphlet very cumbersome to access	1.8 (1.0)
I felt very confident accessing the virtual pamphlet	4.8 (0.5)
I needed to learn a lot of things before I could access the virtual pamphlets	1.3 (0.5)

ON: Ontario.

Items are scored on a Likert-type scale from 1 (strongly disagree) to 5 (strongly agree). Questions were developed using the System of Usability scale: Brooke.^2^

### Qualitative analysis

#### Experiences with the LTC Palliative Toolkit delivered virtually

A total of 14 care partners and 2 residents participated in interviews regarding their experiences with the LTC Palliative Toolkit delivered virtually, with 14 out of the 15 participants from an LTC home in Ontario. One care partner participant was from the LTC home in Saskatchewan. Themes were grouped under the following categories: (a) Need for a palliative approach to care; (b) Palliative pamphlets made access to information easier for families and residents; and (c) Recommendations to improve the experiences with the LTC Palliative Toolkit. “CP” is used to identify quotes from care partners and “RES” for residents. When relevant, the survey data were also compared with the qualitative interview data.

#### Need for a palliative approach to care

##### The pandemic was an impetus for calls to improve a palliative approach to care

Participants voiced that the COVID-19 pandemic disproportionately affected residents, care partners, and staff in LTC compared to the general Canadian population. One care partner shared not being able to witness end-of-life care in person for their loved one due to visitor restrictions. Another care partner discussed the need to always have a formal intervention like the LTC Palliative Toolkit available to ensure that a palliative approach to care can be readily applied. Care partners perceived that a palliative approach to care offers comfort and peace for both residents and care partners. One care partner noted that a palliative approach to care should be about “*keeping people comfortable. Not being drastic. . .Supporting families and the client during the process*” (FAM_022). Care partners and residents reported a greater awareness of the need to improve palliative care early on for residents and to ensure that their care partners are never restricted from them.

I think you have to look at this process and in some ways it’s an end-of-life care even if it’s not you know going to happen next week, next year or whatever and that time is just a short window of what time these people have with their families. The world is so restricted with COVID and so many losses. And this was terrible, this goes on our families and our you know residents in care because they’ve all regressed huge and lost a lot and it’s. . .there’s no way that can come back. You know that is gone and to them I don’t know what is worse, the fear of getting COVID or the fear of not seeing your family or not understanding. (CP_002)

##### Care partners’ palliative approach to care readiness outpaced palliative approach to care literacy

Some care partners did not know what a palliative approach meant until they participated in this study and took part in PCCs. This finding contrasted with the survey data presented in Table 2 as care partners were found to have high values at baseline with regard to feeling prepared for the end-of-life. The difference may be due to the self-reported data provided on the survey, and care partners may have thought they understood a palliative approach, but many knowledge gaps were evident during the interview. One care partner wondered if a palliative approach in LTC would be similar to a previous experience in hospital.

I really didn’t know very much [about palliative care]. I had to deal with it with my dad when he was in the hospital. So, I don’t know if it’s the same thing sort of thing . . . Like my mother now I don’t know what it’s going to be like. She has Dementia. (CP_005)

Another care partner initially associated palliative care with an individual going into hospice care. Care partners perceived the need for more training and support in a palliative approach for care partners. They felt that they had difficulty distinguishing between evidence and common myths. Car partners were having particular challenges translating a palliative approach to care to the kinds of slowly progressing conditions often seen in LTC, such as frailty and dementia. Being informed about what a palliative approach means and how it informs resident care can make it easier for care partners to discuss advance care planning for residents and address the shock and sadness that comes with resident death.

#### Palliative pamphlets made access to information easier for families and residents

##### Easy to navigate and accessible

Care partners reported that the palliative pamphlets were easy to access by clicking on a link that was sent via email. Care partners perceived that locating pertinent information was simple. “*It was relatively easy. I like the fact that you had coloured the headlines, so you knew what you were looking at. It’s all on one page, like virtually. . .it was easy to follow. No issues there*” (CP_007). The pamphlets also appealed to different learning styles as there were links to videos and graphs included as well as written information. Some care partners perceived that older adults including residents who were not familiar with technology may, however, have a difficult time accessing and navigating the pamphlet. Paper copies of pamphlets might be more suitable for this group.

##### Information provided was helpful

Care partners perceived that the pamphlets offered information that was helpful in addressing their knowledge gaps through practical information about disease trajectories, planning for residents, and empowering them to make treatment decisions. These findings aligned with the survey data presented in Table 3 as on average respondents agreed to strongly agreed that the information was helpful. The visuals provided in the information materials, including graphs, videos, and photos, were effective. One care partner mentioned that they liked that the pamphlets were specific to diseases.

It is relevant because my mom does have Advanced Dementia. She’s not in end-of-life stages yet. But for me it was relevant because it’s a matter of looking and answering some questions. What to expect. That I found very helpful. (CP_007)

Care partners perceived that the pamphlets supported their advance care planning and helped them to set realistic plans of care for their loved ones. The information helped care partners set realistic goals which in turn helped them address comfort and psychosocial needs. One care partner shared that after having read the pamphlets they were better able to understand advanced care planning and reaffirm their beliefs. “*I don’t want people doing heroics on my mother. . .once it comes end-of-life, manage any pain or symptom management and any spiritual management that she needs*” (CP_008).

#### Recommendations to improve implementation and utilization of the LTC Palliative Toolkit

##### Calls for stronger government regulation of LTC, including a focus on a palliative approach to care

Families and residents perceived the need to improve care delivery in LTC, including a greater emphasis on a palliative approach to care. The ability to scale up the use of resources included in the Toolkit was perceived by participants as dependent on the agenda of politicians.

I guess the question is what are the standards given by the political representatives for long term care? What do they have implemented? Do they have something implemented for palliative care? Or do they have a clue or are they leaving it up to individual homes to develop their own based on some kind of strategy? (CP_008)

Care partners perceived the need to have political leaders identify improvements in LTC as a priority.

Well, I think there should be a national standard and I think the federal government should be involved in that. Although the ministry of long-term care and whatever is provincial, but I do think that would be the optimal way in the long run. (CP_002)

One care partner suggested having LTC home inspectors monitor the appropriate use of the Toolkit.

This is a Toolkit. You can go in a home and say you’re not doing this and you’re not doing that. They need inspectors coming in on a regular basis. I don’t know if it’s provincial inspectors or federal or both, but they need inspections done. Inspectors have the toolkit and then people highlight what the main problems are. (CP_006)

##### Improving a palliative approach to care literacy

Care partners and residents perceived that there is a need to enhance a palliative approach to care literacy. They perceived that by promoting the Toolkit to a broader audience such as members of the public, this could enhance its reach and help people better understand a palliative approach to care. One care partner discussed how it is important to have members of the public start thinking about a palliative approach to care as they may be affected by this in their own lives in the near future.

I think knowledge is power right? A lot of people don’t know anything about it [a palliative approach to care], nor do they want to know anything about it. I guess it doesn’t affect them. So, if it doesn’t affect them I don’t have to care about it. Whereas in two years when it might affect them, they’re thinking hmmm, I guess I should have paid attention to that. (CP_007)

Care partners perceived that introducing the Toolkit at family councils and finding advocates with lived experience could be helpful.

I guess what I’d say there is I’m sure there’s a ton of people out there that have gone through death and dying with a loved one. You know myself specifically. If I understood this toolkit and I felt like I really understood it and could bring it to people, you know I’d be quite willing to talk about that and my experiences. And how you know this toolkit, how it could help with the experiences you’re going through. Just have advocates that way. I think, nothing better than someone who has really done the job you know? (CP_008)

Care partners and residents recommended creating partnerships between the research team and organizations with strong voices and well-known presence such as the Canadian Medical Association, the Registered Nurses Association of Ontario, and the Alzheimer’s Society to lead to a greater awareness of a palliative approach to care. Some of the care partners perceived the need to include more resources and strategies in the Toolkit such as more bereavement resources, how to connect with the Alzheimer’s Society, and how to designate a power of attorney. One care partner suggested including information on a local bereavement support program for individuals who have lost a spouse.

Well because you highlighted Bereaved Families, I just wondered if you should highlight Widows to Widows which was a program run through the [organization]. Especially for people who had lost spouses. So that was a really good program for that. And I thought that would probably really apply to long term care. Since there’s a lot of husbands or wives that are there. (CP_022)

Care partners and residents perceived that the improvement in literacy of a palliative approach to care depended on the involvement and education of staff of all disciplines, including those in administration to those in non-clinical roles such as housekeepers. Buy-in of all staff was necessary to support care partners’ residents. One resident explained that staff need to be provided with information that is easy to understand:

Long term care, there is so much variance in different people. Look at the difference in people that we have just on this floor. Like how do you tell a PSW you have to look after these people? So, in the implementation of this program too like you have to realize it should really be a program where you’re giving it to people of a certain caliber and then it should be like going from grade one to grade five. Like written down for anyone to understand. (RES_002)

By providing tailored resources like the Toolkit to staff, they could be better equipped to support a palliative approach to care for residents and care partners.

##### Providing pamphlets in an accessible format

Care partners had many recommendations to make the pamphlets more accessible. Several care partners perceived that people living with age-related vision change, or other visual impairment may have difficulty accessing and navigating the pamphlets. They suggested offering access to a larger font online and creating large-font hard copy resources.

For the elderly you would need large print. Hopefully some of them could read. I don’t know when they get to a certain age you know. . .like I said, my mother has Dementia. She could read it but then forget. But at least it’s there, it could be nice. I don’t even know if they like to read. . .but if it is handy and if they could see it and read it in their own language. . .makes them think about it right? (CP_005)

Care partners also recommended making the pamphlets accessible in multiple languages given Canada’s multicultural context.

## Discussion

In this multiple methods study, we described the experiences of care partners and residents with specific components of the LTC Palliative Toolkit consisting of the pamphlets and PCCs. This study was the first to describe the experiences with the Toolkit to support a palliative approach to care during the later waves of the COVID-19 pandemic (April 2021–January 2023) from the perspectives of care partners and residents. This was an important issue to address to deal with the aftermath of the early periods of the pandemic as the care needs of residents continued to be impacted by the pandemic. This study also addresses an understudied area consisting of the evaluation of interventions to support caregivers with advanced care planning. Key findings were: (a) PCCs may help care partners be better prepared to cope with illness, dying, and grief; (b) the need for a palliative approach to care became increasingly apparent during the pandemic; (c) LTC staff of all disciplines need to be involved in supporting a palliative approach to care; and (d) government action is needed to improve uptake and sustainability of strategic initiatives to improve palliative care in LTC.

Although care partners in this current study perceived that they were well prepared for the end-of-life in all domains of the Caring Ahead survey (i.e., dementia knowledge, actions, emotions and support needs, and overall preparedness) prior to PCCs, there was a small statistically significant improvement in terms of being prepared for dying, grief processes, and being able to identify a confidant for emotional support post-conference. This finding suggests that PCCs are supporting care partners with a better understanding of what might happen after their relative’s death, including their own grief process. PCCs also seemed to help families take the step to identify someone they could turn to for emotional support to talk about the meaning of illness and dying after the PCC. These quantitative findings align with the interview data as care partners perceived that information provided to them at the PCCs and within the pamphlets was helpful. In a pandemic context, feelings of grief have been found to be enhanced in care partners as they are unsure of how a disease will progress or how LTC policy changes will affect them.^29^

Similar to our survey findings regarding the effects of the PCCs on care partners’ preparedness for end-of-life, Parker et al. found that structured, interdisciplinary PCCs in LTC addressed multiple components of palliative and end-of-life care, and 90% of families perceived that issues related to end-of-life care planning, spiritual care, and pain and symptom management were adequately addressed.^30^ The authors reported that families had a better understanding of the current and future care of their family members in LTC, however, staff needed to spend more time addressing issues related to care processes and the role of family.^30^ Durepos et al. found that family grief, emotions, and loss and bereavement were poorly discussed at PCCs.^31^ In contrast, our survey findings revealed that emotional and support needs improved as a result of conferences. Clear communication regarding what to expect at end-of-life by LTC staff is essential to support care partners’.^32^ These findings reveal the need to support care partners in LTC to help them reflect on their current situation and future outcomes.

In this study, we found that the COVID-19 pandemic created greater awareness for care partners and residents in LTC regarding the need for a palliative approach to care provided through specific components of the LTC Palliative Toolkit, as many care partners were experiencing the sudden decline and death of their loved ones. Some care partners lacked knowledge of a palliative approach in LTC and benefitted from learning about the palliative approach to care, especially through the informational pamphlets provided. Therefore, a LTC Palliative Toolkit appears to have met a gap in care for residents and families. Although there are comprehensive international COVID-19 guidelines for a palliative approach in LTC, many of these have limitations and fail to cover important aspects such as care partner support and staff education.^33^

With strict measures put in place globally to contain the spread of COVID-19, residents were dealing with the effects of social isolation including an increased risk of mortality.^34^ In this context, palliative interventions were found to help staff make resident-centered decisions when faced with ethical challenges related to adhering to measures to contain the virus and the devastating effects of separating residents from their care partners.^34^ Older adults are most vulnerable to sudden health deterioration as a result of contracting COVID-19 and may not have all of their palliative needs met by LTC staff when resources are stretched.^6^ When staff are faced with high demands, essential care including bereavement support may be missed.^35^ In alignment with our current study, Bolt et al. emphasized the importance of providing information to residents and families early on in LTC and documenting wishes in advance, which becomes even more useful to refer to when a pandemic is occurring.^6^ Findings from these previous studies resonate with the current study regarding the need to provide accessible and clear information for families and residents about a palliative approach and what to expect in LTC with options for virtual formats. Outcomes were consistent across the three provinces suggesting that the Toolkit can be implemented in various settings despite the variations in LTC homes or resident population.

The success of the LTC Palliative Toolkit was perceived by care partners and residents in the current study as requiring the involvement of educated LTC staff belonging to a variety of professional disciplines including registered staff and PSWs. This could help ensure that messaging regarding a palliative approach is consistent across disciplines. In support of our proposed interdisciplinary palliative approach to care, Leclerc et al. found that attitudes toward death, end-of-life, palliative care, and interdisciplinary practice were more favorable in nurses, physicians, rehabilitation staff, and managers compared to nursing assistants and volunteers.^36^ To fully embrace an inclusive palliative approach, non-clinical staff could benefit increased literacy of a palliative approach to care as they interact frequently with residents and families and become emotionally attached.^37^ Coffey et al. similarly identified multidisciplinary engagement at all levels as a facilitator for delivering palliative interventions for residents with dementia in LTC.^38^

One of the barriers identified by care partners and residents in the current study was that there is a need for provincial governments to enforce LTC standards to support a palliative approach. In Ontario, Canada, the government created the Fixing LTC Act in 2021, which provides vague stipulations regarding what is needed to support a palliative approach in LTC, leaving LTC homes to decide how a palliative philosophy is to be integrated.^39^ Policymakers recognized that the pandemic was an impetus for the introduction of supportive palliative care legislation for LTC, however, there is more that needs to be done including oversight to ensure strategic implementation and results of a palliative approach to care, attention to measures for residents and families, and greater attention to supporting adaptation in Canada’s multicultural context. Researchers can support these initiatives by contributing metrics or evaluating strategic programs or changes to operationalize policy.

### Implications for practice, policy, and research

With regards to practice implications, care partners and residents perceived the importance of interdisciplinary LTC staff discussing a palliative approach to care with them. LTC homes should consider implementing dedicated time for care planning discussions in the form of a PCC to reduce stress, anxiety, and unnecessary hospitalizations for residents^30^ or during existing family care conferences. Families and residents should be provided with written resources such as pamphlets and opportunities for meaningful discussions with LTC staff using standardized tools that avoid a “tick-box” approach.^40^ From a policy perspective, access to care and information about a palliative approach to care should be available for care partners and residents.^41^ In response to the COVID-19 pandemic restrictions limiting in-person visits, we offered virtual copies of informational pamphlets for care partners. It is important to ensure that care partners and residents have the necessary equipment to access information virtually, and LTC homes should offer education on using technology for care partners and residents. Future research could consist of a multi-jurisdictional randomized controlled trial with a large sample size to further evaluate the effects of the LTC Palliative Toolkit for families and residents across Canada. The perspectives of staff regarding the effects of the Toolkit will be described in a forthcoming paper.

### Strengths and limitations

This study used a multiple methods approach to explore the user experience of the LTC Palliative Toolkit across three Canadian provinces, and data were collected through surveys and interviews. Only one home from each province was included in the study due to time and funding constraints. Although this research offers a fairly detailed inside look at the experiences of a diverse cross-section of Canadians as related to the implementation of a palliative approach in LTC during the pandemic, given the small number of homes and people represented, additional research would be needed to more accurately ascertain the experience in each province or across Canada. The limitations, most likely due to the pandemic context, included the small sample size, low number of resident participation, lack of validation and pilot testing of the bereavement pamphlet survey, lack of staff perspectives, and uneven numbers of interviews and surveys completed across provinces as certain aspects of the study were not conducted in some provinces. A non-random convenience sample and selection bias were also limitations. Some selection bias occurred through reliance on a convenience sample, as staff approached care partners and residents to inquire about study participation; additionally, this research did not include non-English speaking care partners and residents. Due to an imbalance in the representation of care partners and residents, results more strongly represent the care partner experience and much less the resident experience. While the pre/post analysis showed significance on the measure of the emotion and support needs, indicating sufficient power to detect the observed effect size in that domain, the low response rate (particularly at post-intervention) is likely to have compromised statistical power to detect smaller effects.

## Conclusion

In this study, we described the perspectives and user experiences of care partners and residents on specific components of the LTC Palliative Toolkit implemented during the COVID-19 pandemic. Care partners and residents perceived that the need for the Toolkit became even more pronounced during the pandemic due to the toll that COVID-19, and the associated requirement for care partner restrictions in LTC, had on the overall well-being of residents. They found it useful to have a LTC Palliative Toolkit available virtually so that they could access information at their convenience and be prepared and informed about what lay ahead for their loved one in LTC. Therefore, with support from policymakers and adoption by LTC homes, the LTC Palliative Toolkit could be a valuable intervention that supports improvement of the delivery of a palliative approach to care in LTC.

## Supplemental Material

sj-docx-1-pcr-10.1177_26323524251393344 – Supplemental material for Experiences of care partners and residents with the Long-Term Care Palliative Toolkit during the COVID-19 pandemic: A multiple methods study Supplemental material, sj-docx-1-pcr-10.1177_26323524251393344 for Experiences of care partners and residents with the Long-Term Care Palliative Toolkit during the COVID-19 pandemic: A multiple methods study by Marie-Lee Yous, Rose McCloskey, Abigail Wickson-Griffiths, Donny Li, Vanessa Maradiaga Rivas, Amit Arya, Sheila A. Boamah, Maureen Dobbins, Pamela Durepos, Paulette V. Hunter, Sarah Neil-Sztramko, Henry Siu, Tamara Sussman, Genevieve Thompson and Sharon Kaasalainen in Palliative Care and Social Practice

sj-docx-2-pcr-10.1177_26323524251393344 – Supplemental material for Experiences of care partners and residents with the Long-Term Care Palliative Toolkit during the COVID-19 pandemic: A multiple methods study Supplemental material, sj-docx-2-pcr-10.1177_26323524251393344 for Experiences of care partners and residents with the Long-Term Care Palliative Toolkit during the COVID-19 pandemic: A multiple methods study by Marie-Lee Yous, Rose McCloskey, Abigail Wickson-Griffiths, Donny Li, Vanessa Maradiaga Rivas, Amit Arya, Sheila A. Boamah, Maureen Dobbins, Pamela Durepos, Paulette V. Hunter, Sarah Neil-Sztramko, Henry Siu, Tamara Sussman, Genevieve Thompson and Sharon Kaasalainen in Palliative Care and Social Practice

sj-docx-3-pcr-10.1177_26323524251393344 – Supplemental material for Experiences of care partners and residents with the Long-Term Care Palliative Toolkit during the COVID-19 pandemic: A multiple methods study Supplemental material, sj-docx-3-pcr-10.1177_26323524251393344 for Experiences of care partners and residents with the Long-Term Care Palliative Toolkit during the COVID-19 pandemic: A multiple methods study by Marie-Lee Yous, Rose McCloskey, Abigail Wickson-Griffiths, Donny Li, Vanessa Maradiaga Rivas, Amit Arya, Sheila A. Boamah, Maureen Dobbins, Pamela Durepos, Paulette V. Hunter, Sarah Neil-Sztramko, Henry Siu, Tamara Sussman, Genevieve Thompson and Sharon Kaasalainen in Palliative Care and Social Practice
