# Supplementary material for: Experiences of care partners and residents with the Long-Term Care Palliative Toolkit during the COVID-19 pandemic: A multiple methods study
Source: Palliat Care Soc Pract. 2025 Nov 12;19:26323524251393344. doi: 10.1177/26323524251393344 (PMC12612540; doi:10.1177/26323524251393344)
Supplement: sj-docx-1-pcr-10.1177_26323524251393344 – Supplemental material for Experiences of care partners and residents with the Long-Term Care Palliative Toolkit during the COVID-19 pandemic: A multiple methods study [file sj-docx-1-pcr-10.1177_26323524251393344.docx]

**Bereavement Pamphlet Survey for Family Members**

*You are being invited to complete the survey because you have read at least one of the pamphlets focused on grief and loss. Your answers are very valuable in the evaluation of these pamphlets.* *By completing this survey, you are agreeing to have your confidential responses used for research purposes. Your responses to the survey will help the research team learn more about your feedback regarding the pamphlet(s) you have read. If you have any further questions about the survey, please do not hesitate to contact a member of the research team.*

1. How did you receive the pamphlet(s)?

- From a staff member at the long-term care home
- From a display board at the long-term care home
- Other, please specify: ______________________________________

1. Did you read the pamphlet on *Grief and Loss* (purple)?

□ Yes (please complete next question) □ No (skip to question 4)

1. The following questions ask about the content of the *Grief and Loss* pamphlet. Please read each statement and circle the option that best fits how you feel about each pamphlet.

| 1 = Strongly Disagree  2= Disagree  3 = Neutral  4 = Agree  5 = Strongly Agree | Strongly Disagree | Disagree | Neutral | Agree | Strongly Agree |
| --- | --- | --- | --- | --- | --- |

| **The information in the pamphlet on “Grief and Loss” (purple)** | | | | | |
| --- | --- | --- | --- | --- | --- |
| The information in this pamphlet was presented clearly. | 1 | 2 | 3 | 4 | 5 |
| The information in this pamphlet was easy to understand. | 1 | 2 | 3 | 4 | 5 |
| The information in this pamphlet was upsetting or distressing. | 1 | 2 | 3 | 4 | 5 |
| The information in this pamphlet was helpful. | 1 | 2 | 3 | 4 | 5 |
| The information in this pamphlet was meaningful and relevant. | 1 | 2 | 3 | 4 | 5 |
| The information in this pamphlet was reassuring. | 1 | 2 | 3 | 4 | 5 |
| The pamphlet had sections that I felt were not important. | 1 | 2 | 3 | 4 | 5 |
| The pamphlet provided very helpful resources. | 1 | 2 | 3 | 4 | 5 |
| Some of the information in this pamphlet was new to me | 1 | 2 | 3 | 4 | 5 |

1. Did you read the pamphlet on *Resources on Grief, Bereavement and Loss* (green)?

□ Yes (please complete next question) □ No (skip to question 6)

1. The following questions ask about the content of the *Resources on Grief, Bereavement and Loss* pamphlet. Please read each statement and circle the option that best fits how you feel about each pamphlet.

| 1 = Strongly Disagree  2= Disagree  3 = Neutral  4 = Agree  5 = Strongly Agree | Strongly Disagree | Disagree | Neutral | Agree | Strongly Agree |
| --- | --- | --- | --- | --- | --- |

| **The information in the pamphlet on “Resources on Grief, Bereavement, and Loss” (green)** | | | | | |
| --- | --- | --- | --- | --- | --- |
| The information in this pamphlet was presented clearly. | 1 | 2 | 3 | 4 | 5 |
| The information in this pamphlet was easy to understand. | 1 | 2 | 3 | 4 | 5 |
| The information in this pamphlet was upsetting or distressing. | 1 | 2 | 3 | 4 | 5 |
| The information in this pamphlet was reassuring. | 1 | 2 | 3 | 4 | 5 |
| The information in this pamphlet was helpful. | 1 | 2 | 3 | 4 | 5 |
| The information in this pamphlet was meaningful and relevant. | 1 | 2 | 3 | 4 | 5 |
| The pamphlet had sections that I felt were not important. | 1 | 2 | 3 | 4 | 5 |
| The pamphlet provided very helpful resources. | 1 | 2 | 3 | 4 | 5 |
| Some of the information in this pamphlet was new to me | 1 | 2 | 3 | 4 | 5 |

1. Did you read the pamphlet on *What to do after a death* (Blue)?

□ Yes (please complete next question) □ No (skip to question 8)

1. The following questions ask about the content of the *What to do after a death* pamphlet. Please read each statement and circle the option that best fits how you feel about each pamphlet.

| 1 = Strongly Disagree  2= Disagree  3 = Neutral  4 = Agree  5 = Strongly Agree | Strongly Disagree | Disagree | Neutral | Agree | Strongly Agree |
| --- | --- | --- | --- | --- | --- |

| **The information in the pamphlet on “What to Do After a Death” (blue)** | | | | | |
| --- | --- | --- | --- | --- | --- |
| The information in this pamphlet was presented clearly. | 1 | 2 | 3 | 4 | 5 |
| The information in this pamphlet was easy to understand. | 1 | 2 | 3 | 4 | 5 |
| The information in this pamphlet was upsetting or distressing. | 1 | 2 | 3 | 4 | 5 |
| The information in this pamphlet was helpful. | 1 | 2 | 3 | 4 | 5 |
| The information in this pamphlet was meaningful and relevant. | 1 | 2 | 3 | 4 | 5 |
| The information in this pamphlet was reassuring. | 1 | 2 | 3 | 4 | 5 |
| The pamphlet had sections that I felt were not important. | 1 | 2 | 3 | 4 | 5 |
| The pamphlet provided very helpful resources. | 1 | 2 | 3 | 4 | 5 |
| Some of the information in this pamphlet was new to me | 1 | 2 | 3 | 4 | 5 |

1. The following questions ask about your comfort level when reading the pamphlets and the extent to which the pamphlets helped or might help you in your grief/ bereavement.

| 1 = Strongly Disagree  2= Disagree  3 = Neutral  4 = Agree  5 = Strongly Agree | Strongly Disagree | Disagree | Neutral | Agree | Strongly Agree |
| --- | --- | --- | --- | --- | --- |

| Reading the pamphlets made me feel supported. | 1 | 2 | 3 | 4 | 5 |
| --- | --- | --- | --- | --- | --- |
| Reading the pamphlets helped me to understand my feelings. | 1 | 2 | 3 | 4 | 5 |
| Reading the pamphlets helped me to feel help was available if needed. | 1 | 2 | 3 | 4 | 5 |
| Reading the pamphlets helped to clarify the actions I may need to take. | 1 | 2 | 3 | 4 | 5 |
| Reading the pamphlets helped to clarify where I can go for additional support. | 1 | 2 | 3 | 4 | 5 |

1. Please check off your gender identity: ❒Male ❒Female ❒Other
2. What is your age in years? __________
3. What is your relationship to the resident? :

❒ Spouse

❒ Child

❒ Sibling

❒ Friend

❒ Other (please describe): _________________________________.

1. Approximately how long has your family member/friend lived in this LTC home**?**

_____years ________months

1. Is your family member/friend still alive?

❒ Yes ❒ No

1. Is your family member/friend receiving end-of-life care now?

❒ Yes ❒ No ❒ Don’t know

1. Which of the following best describes your current situation?

- I am a family member/friend of a resident who is in a stable health condition
- I am a family member/friend of a resident who is near end-of-life
- I am a family member/friend of a resident who has recently died

**Note.** Bereavement pamphlets survey
