# Supplementary material for: Experiences of care partners and residents with the Long-Term Care Palliative Toolkit during the COVID-19 pandemic: A multiple methods study
Source: Palliat Care Soc Pract. 2025 Nov 12;19:26323524251393344. doi: 10.1177/26323524251393344 (PMC12612540; doi:10.1177/26323524251393344)
Supplement: sj-docx-2-pcr-10.1177_26323524251393344 – Supplemental material for Experiences of care partners and residents with the Long-Term Care Palliative Toolkit during the COVID-19 pandemic: A multiple methods study [file sj-docx-2-pcr-10.1177_26323524251393344.docx]

***System Usability Scale* for Care Partners**

© Digital Equipment Corporation, 1986.

Strongly Strongly

disagree agree

1. I think that I would like to use the virtual pamphlet frequently

|  |  |  |  |  |
| --- | --- | --- | --- | --- |

| 1 | 2 | 3 | 4 | 5 |
| --- | --- | --- | --- | --- |
|  |  |  |  |  |
| 1 | 2 | 3 | 4 | 5 |
|  |  |  |  |  |
| 1 | 2 | 3 | 4 | 5 |
|  |  |  |  |  |
| 1 | 2 | 3 | 4 | 5 |

1. I found the virtual pamphlet unnecessarily complex
2. I thought the virtual pamphlet was easy to read

1. I think that I would need the support of a technical person to be able to access the virtual pamphlet

|  |  |  |  |  |
| --- | --- | --- | --- | --- |
|  |  |  |  |  |
| 1 | 2 | 3 | 4 | 5 |

1. I would imagine that most people would learn to access the virtual pamphlet very quickly

|  |  |  |  |  |
| --- | --- | --- | --- | --- |

| 6. I found the virtual pamphlet very cumbersome to access | 1 | 2 | 3 | 4 | 5 |
| --- | --- | --- | --- | --- | --- |
| 7. I felt very confident accessing the virtual pamphlet |  |  |  |  |  |
|  | 1 | 2 | 3 | 4 | 5 |
| 8. I needed to learn a lot of things before I could access the virtual pamphlets |  |  |  |  |  |
|  | 1 | 2 | 3 | 4 | 5 |

**Note.** Information pamphlet survey developed from: Brooke J. SUS-A quick and dirty usability scale. Usability evaluation in industry. 1996 Jun 11;189(194):4-7.
