# Supplementary material for: Experiences of care partners and residents with the Long-Term Care Palliative Toolkit during the COVID-19 pandemic: A multiple methods study
Source: Palliat Care Soc Pract. 2025 Nov 12;19:26323524251393344. doi: 10.1177/26323524251393344 (PMC12612540; doi:10.1177/26323524251393344)
Supplement: sj-docx-3-pcr-10.1177_26323524251393344 – Supplemental material for Experiences of care partners and residents with the Long-Term Care Palliative Toolkit during the COVID-19 pandemic: A multiple methods study [file sj-docx-3-pcr-10.1177_26323524251393344.docx]

**Caring Ahead: Preparing for End-of-Life in Dementia questionnaire**

Instructions:

Caring for a family member or friend with dementia can be very challenging, especially as end-of-life approaches. This questionnaire asks sensitive questions to help understand the knowledge, actions, communication and support you have to prepare for the death of your family member or friend. Please circle one number to show how strongly you agree or disagree with each question statement. Please take your time and reach out for emotional support when needed. Please share your responses with your health care provider.

|  | **Dementia Knowledge Preparations** | **Strongly Strongly**  **Disagree Agree** | | | | | | |
| --- | --- | --- | --- | --- | --- | --- | --- | --- |
| 1. | I believe I understand my family member’s current health status. | 1 | 2 | 3 | 4 | 5 | 6 | 7 |
| 2. | I believe I know what changes to expect as dementia advances, for example: swallowing, eating difficulties. | 1 | 2 | 3 | 4 | 5 | 6 | 7 |
| 3. | I understand I may be faced with decisions such as: transferring to hospital, treating infections. | 1 | 2 | 3 | 4 | 5 | 6 | 7 |
| 4. | I have learned what the dying process may be like for my family member. | 1 | 2 | 3 | 4 | 5 | 6 | 7 |
| 5. | I understand that dementia (and Alzheimer’s) is a condition that leads to death. | 1 | 2 | 3 | 4 | 5 | 6 | 7 |

|  | **Communication Preparations** | **Strongly Strongly Disagree Agree** | | | | | | |
| --- | --- | --- | --- | --- | --- | --- | --- | --- |
| 6. | I believe I know what treatments my family member would choose. | 1 | 2 | 3 | 4 | 5 | 6 | 7 |
| 7. | I have shared my family member’s wishes with health care providers, for example: focusing on living longer, maximizing comfort. | 1 | 2 | 3 | 4 | 5 | 6 | 7 |
| 8. | Health care providers and I have discussed our end-of-life care preferences for my family member, for example: location, pain management. | 1 | 2 | 3 | 4 | 5 | 6 | 7 |
| 9. | I have discussed end-of-life care preferences with the important people in my family member’s life, for example: friends, family. | 1 | 2 | 3 | 4 | 5 | 6 | 7 |

|  | **Action Preparations** | **Strongly Strongly**  **Disagree Agree** | | | | | | |
| --- | --- | --- | --- | --- | --- | --- | --- | --- |
| 10. | I am making the most of my time with my family member, for example: by saying what I need to say to them. | 1 | 2 | 3 | 4 | 5 | 6 | 7 |
| 11. | I am helping my family member with activities they would want near end-of-life, for example: seeing old friends. | 1 | 2 | 3 | 4 | 5 | 6 | 7 |
| 12. | I am spending time reflecting on my family member’s life, for example: by sharing stories. | 1 | 2 | 3 | 4 | 5 | 6 | 7 |
| 13. | I believe I can carry out my responsibilities, for example: decision-maker, companion, advocate. | 1 | 2 | 3 | 4 | 5 | 6 | 7 |
| 14. | My family member’s affairs are in order, for example: decision-maker, will, banking, credit cards, insurance. | 1 | 2 | 3 | 4 | 5 | 6 | 7 |
| 15. | After-death services are arranged for my family member such as: burial or cremation. | 1 | 2 | 3 | 4 | 5 | 6 | 7 |
| 16. | I am thinking about memorials appropriate for our family such as: gathering, funeral, celebration, private time. | 1 | 2 | 3 | 4 | 5 | 6 | 7 |

|  | **Emotions and Support Needs Preparations** | **Strongly Strongly**  **Disagree Agree** | | | | | | |
| --- | --- | --- | --- | --- | --- | --- | --- | --- |
| 17. | I am aware of after-death policies I will need to follow, for example: moving belongings in long-term care. | 1 | 2 | 3 | 4 | 5 | 6 | 7 |
| 18. | I understand what my grief process may be like after my family member has died. | 1 | 2 | 3 | 4 | 5 | 6 | 7 |
| 19. | I have someone to go to for emotional support about my family member’s health. | 1 | 2 | 3 | 4 | 5 | 6 | 7 |
| 20. | I have someone I can talk to about the meaning of illness or dying. | 1 | 2 | 3 | 4 | 5 | 6 | 7 |

| **Overall Preparedness** | **Not Prepared**  **Prepared As Much**  **At All As Possible** | | | | | | |
| --- | --- | --- | --- | --- | --- | --- | --- |
| *If your loved one were to die soon, how prepared would you be for his/her death? | 1 | 2 | 3 | 4 | 5 | 6 | 7 |

Proposed Scoring Instructions: Sum items to create a total score for each subscale. Divide the subscale total by the number of items to calculate an average subscale score.

| Dementia Knowledge (Items 1-5) | Total Score out of 35: ________________  Average Score out of 7: _______________ |
| --- | --- |
| Communication (Items 6-9) | Total Score out of 28: ________________  Average Score out of 7: _______________ |
| Actions (Items 10-16) | Total Score out of 49: ________________  Average Score out of 7: _______________ |
| Emotions and Support Needs (17-20) | Total Score out of 28: ________________  Average Score out of 7: _______________ |

**Note.** Caring Ahead Survey from: Durepos, P., Akhtar-Danesh, N., Sussman, T., Ploeg, J., Boerner, K., & Kaasalainen, S. (2021). Evaluation of the Caring Ahead: Preparing for End-of-Life With Dementia Questionnaire. *Journal of the American Medical Directors Association, 22*(10), 2108-2114.
